# Levels of Different Microbial Groups on Inert Surfaces of Poultry Slaughterhouses: Identification Using Matrix-Assisted Laser Desorption Ionization Time-of-Flight and Detection of Extended-Spectrum Beta-Lactamase- and Carbapenemase-Producing Enterobacteria

**DOI:** 10.3390/antibiotics13070587

**Published:** 2024-06-25

**Authors:** Sarah Panera-Martínez, Cristina Rodríguez-Melcón, Daniel Rodríguez-Campos, Nuria Pérez-Estébanez, Rosa Capita, Carlos Alonso-Calleja

**Affiliations:** 1 Department of Food Hygiene and Technology, Veterinary Faculty, University of León, E-24071 León, Spain; 2 Institute of Food Science and Technology, University of León, E-24071 León, Spain

**Keywords:** poultry slaughterhouses, inert surfaces, microbial load, antibiotic resistance

## Abstract

Knowledge of the microbiota present in food processing environments is a significant advance that will allow for better evaluation of the risk of food contamination and a better design of the procedures for sanitization. The levels of microbial group indicators of hygienic quality were determined in different areas of the slaughter lines of two poultry slaughterhouses in the northwest of Spain (22 surfaces in each slaughterhouse were studied). The average microbial levels (cfu/cm^2^) were 2.15 × 10^2^ ± 4.26 × 10^2^ (total aerobic counts, TAC), 1.99 × 10^2^ ± 5.00 × 10^2^ (psychrotrophic microorganisms), 3.10 × 10^0^ ± 1.37 × 10^1^ (enterobacteria), 3.96 × 10^0^ ± 2.55 × 10^1^ (coliforms), 1.80 × 10^−1^ ± 7.79 × 10^−1^ (enterococci), and 1.12 × 10^−1^ ± 3.35 × 10^−1^ (vancomycin-resistant enterococci, VRE). TAC and psychrotrophic microorganisms were the most abundant groups in all samples (*p* < 0.05). The counts of both microbial groups were higher (*p* < 0.05) in samples of Slaughterhouse A than in those of Slaughterhouse B. Microbial loads for the rest of the bacteria were not influenced by the slaughterhouse sampled (*p* > 0.05). All 44 samples showed TAC and psychrotrophic microorganisms. Colonies of the rest of the microbial groups were only found in 26 samples (59.1% of the total). The isolates (one from each sample) were identified with MALDI-TOF and PCR. Gram-negative bacteria (all *Enterobacteriaceae*) were isolated in 23 samples, and Gram-positive bacteria were isolated in 16 (9 *Enterococcus* spp., 2 *Enterococcus* spp. and VRE, 3 VRE, 1 *Enterococcus* spp. and *Listeria* spp., and 1 *Listeria* spp.). The resistance of the strains to 11 (*Enterococcus* spp.) or 17 (*Enterobacteriaceae*) antibiotics was determined (disk diffusion, CLSI), finding an average of 2.05 ± 2.06 resistances per strain (3.46 ± 2.27 if reduced susceptibility reactions are included). A total of 37.3% of the *Enterobacteriaceae* isolates had a gene for resistance to beta-lactam antibiotics (*bla_TEM_*, *bla_CTX-M-15_*, *bla_KPC_*, *bla_CMY__-2_* or *bla_NDM_*). The high prevalence of resistant bacteria and resistance genes highlights the need to establish measures to control the spread of antibiotic resistance in poultry slaughterhouses. The findings of this work could contribute to the design of more effective sanitation procedures.

## 1. Introduction

The high consumption (10.1 kg per capita worldwide and 23.2 kg per capita in Europe) [1] and production (139.2 million tons worldwide and 12.8 million tons in Europe) [2] of poultry meat justifies the importance of guaranteeing that this food is safe for the consumer and has a long shelf life [3,4]. To this end, numerous control measures have been implemented in recent years throughout the entire poultry meat production chain, such as feeding farm animals with prebiotics or vaccinating them against some pathogens [5,6]. However, the degree of contamination of this food with pathogenic and spoilage microorganisms remains high [7]. An important part of this contamination can occur during the dressing of the carcasses in the slaughterhouse through contact with the different facility surfaces [8,9,10]. The degree of food contamination is directly related to the microbial load on surfaces and equipment in processing plants [11]. Cross-contamination is especially high when correct cleaning and disinfection protocols are not followed [12,13].

The problem of microbial contamination of poultry meat is aggravated when microorganisms present antimicrobial resistance (AMR). The spread of AMR poses a serious threat to public health, as the effective treatment of infections is hampered, leading to increased costs for the health system and increased mortality [14,15]. Beta-lactams are the antibiotics of choice for the treatment of many food-borne infections, so it is essential to know and monitor the prevalence of extended-spectrum beta-lactamases (ESBL) in bacteria of food origin [16]. The prevalence of these enzymes in *Enterobacteriaceae* isolates from broilers has been studied in many European countries at the abattoir level and has provided further evidence of chicken meat as a potential zoonotic source of ESBL-producing bacteria [17]. Although there are a large number of genes that encode ESBL enzymes, in Europe, the CTX-M and TEM groups are some of the most frequently detected in food-producing animals [18]. However, recent studies carried out in Spain and Portugal have reported the presence of other resistance genes, such as *bla_NDM-1_* (61.2% of isolates) [19], *bla_CMY-2_* (76.2%) [20]), or *bla_KPC_* (91.0%) [21], in some EBSL-producing *Enterobacteriaceae* isolates.

Given that the presence of high microbial levels in processing environments directly influences the microbiological quality of meat [22,23], comprehensive monitoring of this microbiota is crucial. Total aerobic counts (TAC), psychrotrophs, *Enterobacteriaceae*, coliforms, and enterococci are among the most common microbial groups used in meat and poultry industries as general indicators of processing hygiene, storage quality, and potential shelf life, both in the oxygen atmosphere and in vacuum-packed meat [3]. For the detection and quantification of microorganisms on surfaces and equipment in food industries, analysis techniques have traditionally been used that involve their cultivation in different media (selective and non-selective), with the subsequent isolation and identification of the colonies performed using biochemical or molecular methods such as polymerase chain reaction (PCR) [24]. In the last decade, matrix-assisted laser desorption/ionization (MALDI-TOF) has gained popularity in microbiology laboratories, as it allows for the identification of bacteria in a quick and easy way [25].

One of the strategies to reduce the contamination of poultry meat is to reduce microbial levels on slaughterhouse surfaces and equipment. To achieve this, in the first stage, it is necessary to expand current knowledge about the microbiota of the different areas of the slaughter line, from stunning to cutting. This research has been proposed in this context, the objective of which was to determine the microbial groups present on the surfaces and equipment of two poultry slaughterhouses and to characterize their resistance to antibiotics, emphasizing the presence of extended-spectrum beta-lactamases.

## 2. Results

### 2.1. Microbial Counts

The levels of the different microbial groups studied (total aerobic counts (TAC), psychrotrophs, enterobacteria, coliforms, enterococci, and vancomycin-resistant enterococci (VRE)) are shown in Table 1. The TAC and psychrotrophic microorganisms were the most abundant bacterial groups in all samples (*p* < 0.05), with average values (cfu/cm^2^) of 2.15 × 10^2^ ± 4.26 × 10^2^ and 1.99 × 10^2^ ± 5.00 × 10^2^, respectively. The lowest plate counts were found for VRE, with an average value (cfu/cm^2^) of 1.12 × 10^−1^ ± 3.35 × 10^−1^. No significant differences (*p* > 0.05) were found in terms of the plate counts between the rest of the microbial groups studied. The mean values were 3.10 × 10^0^ ± 1.37 × 10^1^ for enterobacteria, 3.96 × 10^0^ ± 2.55 × 10^1^ for coliforms, and 1.80 × 10^−1^ ± 7.79 × 10^−1^ for enterococci. The TAC and psychrotrophic loads were higher (*p* < 0.05) in samples from Slaughterhouse A (2.90 × 10^2^ ± 5.25 × 10^2^ and 2.14 × 10^2^ ± 3.59 × 10^2^, respectively) than they were in samples from Slaughterhouse B (1.41 × 10^2^ ± 2.91 × 10^2^ and 1.84 × 10^2^ ± 6.18 × 10^2^, respectively). Microbial loads for the rest of the bacterial groups were not influenced by the slaughterhouse sampled (*p* > 0.05).

### 2.2. Isolation and Identification of Microorganisms

One colony from each microbial group, except for TAC and psychrotrophic microorganisms, was isolated from each sample for subsequent identification using MALDI-TOF and PCR. Microorganisms could be isolated from 26 samples (Table 2), representing 59.1% of the total. In 23 of these samples (88.5% of the positive ones and 52.3% of the total), Gram-negative bacteria (all *Enterobacteriaceae*) were isolated, while only Gram-positive bacterial genera were isolated in 16 samples (61.5% of the positive ones and 36.4% of the total). Strains of *Enterococcus* spp. were isolated in 15 of the samples with Gram-positive bacteria, and VRE were found in 5 of them. Colonies with the typical morphology of *Listeria* spp. were found in two samples (A13 and A22; 7.7% of the positive samples and 4.5% of the total).

### 2.3. Phenotypic Resistance to Antibiotics

**Figure 1:**
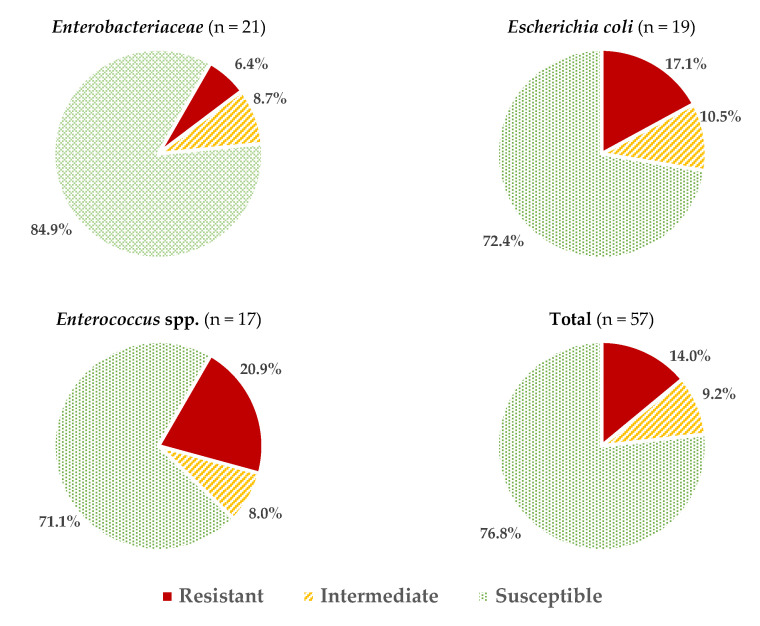
Average percentages of resistant, intermediate (with reduced susceptibility), and susceptible strains in the different microbial groups studied.

Considering all the data obtained for all the microbial groups studied, an average of 2.05 ± 2.06 resistances per strain were found, or 3.46 ± 2.27 if reduced susceptibility reactions are also included. These values vary depending on the microorganism considered (Figure 1), with the isolates of *Enterococcus* spp. and *E. coli* having the highest percentage of resistance (20.9 ± 11.6% and 17.0 ± 15.5%, respectively; these percentages were calculated considering the number of positive reactions (resistance) over the total number of tests carried out (strains × antibiotics). The resistance values (%) for other genera of the family *Enterobacteriaceae* (6.4 ± 6.5%) were significantly lower (*p* > 0.05).

**Figure 2:**
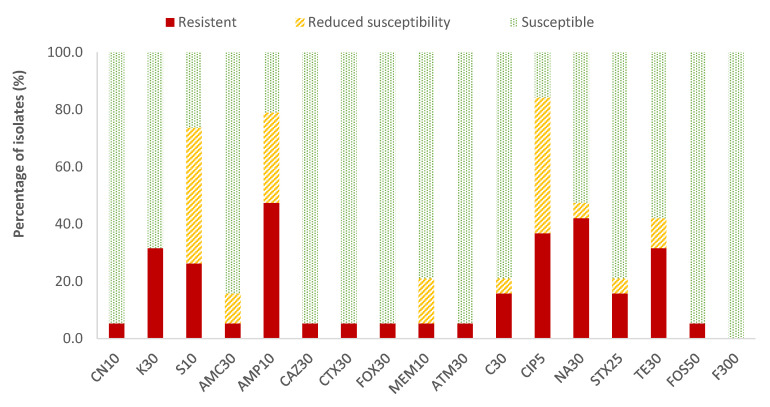
Percentage of resistant *E. coli* strains, with reduced susceptibility or susceptibility to each of the antibiotics tested. Gentamicin, 10 µg (CN10), kanamycin, 30 µg (K30), streptomycin, 10 µg (S10), amoxicillin/clavulanic acid, 30 µg (AMC30), ampicillin, 10 µg (AMP10), ceftazidime, 30 µg (CAZ30), cefotaxime, 30 µg (CTX30), cefoxitin, 30 µg (FOX30), meropenem, 10 µg (MEM10), aztreonam, 30 µg (ATM30), chloramphenicol, 30 µg (C30), ciprofloxacin, 5 µg (CIP5), nalidixic acid, 30 µg (NA30), sulfamethoxazole/trimethoprim, 25 µg (SXT25), tetracycline, 30 µg (TE30), fosfomycin, 50 µg (FOS50), nitrofurantoin, 300 µg (F300).

**Figure 3:**
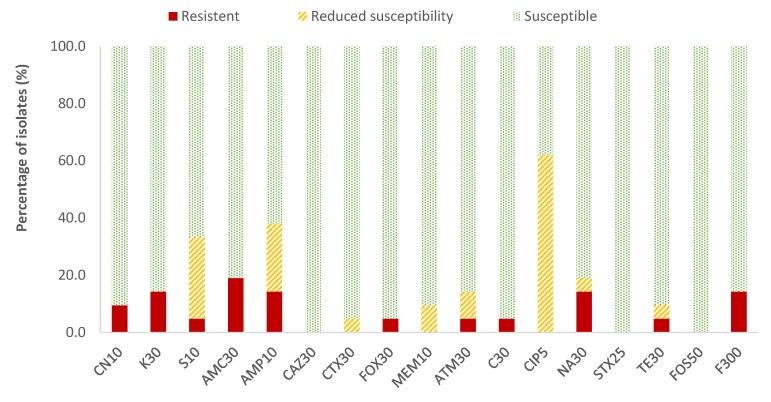
Percentage of resistant *Enterobacteriaceae* strains other than *E. coli*, with reduced susceptibility or susceptibility to each of the antibiotics tested. Gentamicin, 10 µg (CN10), kanamycin, 30 µg (K30), streptomycin, 10 µg (S10), amoxicillin/clavulanic acid, 30 µg (AMC30), ampicillin, 10 µg (AMP10), ceftazidime, 30 µg (CAZ30), cefotaxime, 30 µg (CTX30), cefoxitin, 30 µg (FOX30), meropenem, 10 µg (MEM10), aztreonam, 30 µg (ATM30), chloramphenicol, 30 µg (C30), ciprofloxacin, 5 µg (CIP5), nalidixic acid, 30 µg (NA30), sulfamethoxazole/trimethoprim, 25 µg (SXT25), tetracycline, 30 µg (TE30), fosfomycin, 50 µg (FOS50), nitrofurantoin, 300 µg (F300).

In the case of *Enterobacteriaceae*, 100% susceptibility to ceftazidime (CAZ) sulfamethoxazole/trimethoprim (SXT) and fosfomycin (FOS) was observed, with the exception of *E. coli*, for which there was just one antibiotic (nitrofurantoin, F) to which all strains were susceptible; the resistance percentages of *E. coli* to ceftazidime (CAZ), fosfomycin (FOS) and sulfamethoxazole/trimethoprim (SXT) were 5.3%, 5.3% and 15.8%, respectively. There were no antibiotics for which all *Enterobacteriaceae* strains were resistant to it. For the rest of the antibiotics, in *E. coli* the highest prevalence of resistance was obtained for ampicillin (AMP; 47.4%) and nalidixic acid (NA; 42.1%), while for the rest of the enterobacteria, both antibiotics showed 14.3% of resistances. Considering both resistance and reduced susceptibility, the highest values were obtained in the case of ciprofloxacin (CIP), with resistance prevalences of 84.2% and 61.9% for *E. coli* and for the rest of enterobacteria, respectively, and for ampicillin (AMP), with 78.9% resistance for *E. coli* and 38.1% for the rest of the enterobacteria (Figure 2 and Figure 3).

**Figure 4:**
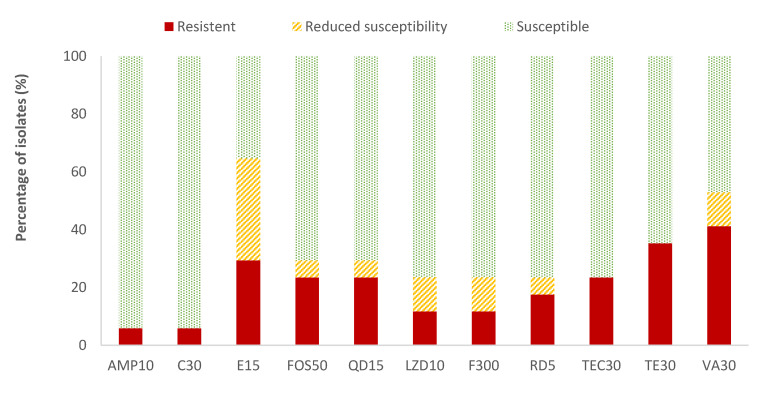
Percentage of *Enterococcus* spp. strains resistant, intermediate (with reduced susceptibility), or susceptible to each of the antibiotics tested. Ampicillin, 10 µg (AMP10), chloramphenicol, 30 µg (C30), erythromycin, 15 µg (E15), fosfomycin, 50 µg (FOS50), quinupristin/dalfopristin, 15 µg (QD15), linezolid, 10 µg (LZD10), nitrofurantoin, 300 µg (F300), rifampicin, 5 µg (RD5), teicoplanin, 30 µg (TEC30), tetracycline, 30 µg (TE30), and vancomycin, 30 µg (VA).

The highest resistance percentages of *Enterococcus* spp. were found for vancomycin (41.2%) and tetracycline (35.3%), while ampicillin (AMP) and chloramphenicol (C) showed the lowest resistant percentages (5.9%). The resistance percentages of the rest of the antibiotics evaluated ranged between 11.8% (for linezolid and nitrofurantoin) and 29.4% (for erythromycin). Considering all the data on the resistance and reduced susceptibility of *Enterococcus* spp., the high values observed for erythromycin (64.7%), vancomycin (52.9%), and tetracycline (35.3%) are notable (Figure 4). *Listeria* spp. was only detected in two samples. Therefore, as not average data could be obtained, the susceptibility testing to antibiotics was not performed to this microorganism.

### 2.4. Detection of Resistance Genes to Beta-Lactam Antibiotics in Enterobacteriaceae

**Figure 5:**
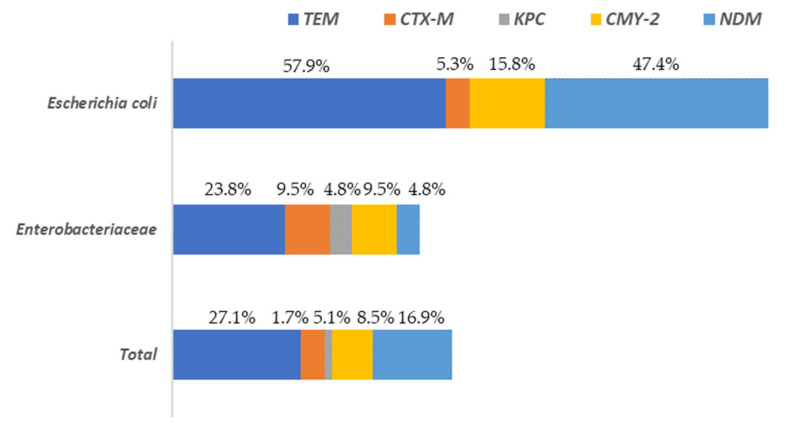
Distribution of beta-lactam antibiotic resistance genes among *Escherichia* and the rest of *Enterobacteriaceae* (other than *E. coli*) isolated on surfaces in both slaughterhouses.

A total of 37.3% of the isolates presented at least one of the resistance genes to the beta-lactam antibiotics analyzed (*bla_TEM_*, *bla_CTX-M_*, *bla_KPC_*, *bla_CMY-2_* or *bla_NDM_*). These percentages varied depending on the microbial group considered, being 78.9% for *E. coli* and 33.3% for the rest of the enterobacteria. The gene with the greatest presence in all *Enterobacteriaceae* isolates, was *bla_TEM_*, followed by *bla_NDM_* and *bla_CMY-2_* (Figure 5).

The highest levels of *bla_TEM_* were found in *E. coli* (57.9%) isolates while for the rest of *Enterobacteriaceae* its prevalence was notably lower (23.8%). The genes *bla_CTX-M_* and *bla_KPC_* were present in a very low percentage of the isolates (5.1% and 1.7%, respectively), with the highest values (9.5% and 4.8%, respectively) observed for the *Enterobacteriaceae* isolates, without taking into account *E. coli*. Regarding to the rest of the genes studied (*bla_CMY-2_* and *bla_NDM_*) the highest values were found in *E. coli* isolates, ranging from 15.8% (*bla_CMY-2_*) to 47.4% (*bla_NDM_*). In the Supplementary Materials (Table S1) the results for all isolates tested for AST and the presence of AMR are shown. A column is also included to identify the sample origin (in which slaughterhouse the surface was found), as well as another for genus identification.

## 3. Discussion

### 3.1. Microbial Counts

The average TAC (2.15 × 10^2^ ± 4.26 × 10^2^ cfu/cm^2^) is similar to those observed by Whyte et al. [26] in three poultry meat production plants in Ireland, who found values between 2.8 × 10^1^ and 1.8 × 10^3^ cfu/cm^2^. Notably, in the present study, 72.7% of the surfaces analyzed had counts greater than 10 cfu/cm^2^ for this microbial group, a percentage that is considered high. The microbiological criteria for inert surfaces in meat industries established in Decision 2001/471/EC indicated that the number of bacteria present on a surface is acceptable if it does not exceed the value of 10 cfu/cm^2^ (TAC) or 1 cfu/cm^2^ (*Enterobacteriaceae*). With values higher than those indicated, the aforementioned standard established the need to implement corrective actions. Although this decision was repealed with effect from 1 January 2006, these microbiological criteria can still be used to evaluate hygiene in meat industries. The TAC values in the present study are considerably higher than those previously obtained by Alonso-Calleja et al. [27] in lamb slaughterhouses, where the percentages of samples with values greater than 10 cfu/cm^2^ varied between 3.1 and 17.1%. In this regard, it should be noted that the differences between slaughterhouses must be interpreted with caution due to the fundamental particularities of one, with regard to the species slaughtered, which present different levels and types of microorganisms.

The mean counts of psychrotrophic microorganisms observed in this study (1.99 × 10^2^ ± 5.0 × 10^2^ cfu/cm^2^) were also similar to those previously described by Whyte et al. [26], who obtained values between 1.4 × 10^1^ and 3.6 × 10^2^ cfu/cm^2^. However, other authors found much higher levels of psychrotrophs as follows: between 2.5 × 10^1^ and 5.0 × 10^7^ cfu/cm^2^ [28] and between 1.9 × 10^3^ and 6.3 × 10^6^ cfu/cm^2^ [29] on surfaces in a bovine slaughterhouse.

At this point in the production chain, it is essential to prevent psychrotrophic microorganisms from adhering to the carcass to produce meat with a long shelf life [20]. The presence of high counts of these microorganisms on food contact surfaces may pose a health risk to consumers due to the possibility of the cross-contamination of meat and, subsequently, their ability to grow and multiply under refrigerated conditions during storage and transportation [30]. Furthermore, psychrotrophic microorganisms include some spoilage bacteria (i.e., *Pseudomonas* or *Brochothrix*), so high counts of these microorganisms can be associated with important economic losses [3].

*Enterobacteriaceae* counts (3.10 × 10^0^ ± 1.37 × 10^1^ cfu/cm^2^) were also similar to those obtained by other authors (between 3.9 × 10^−1^ and 4.2 × 10^1^ cfu/cm^2^) [26] on poultry slaughterhouse surfaces. Furthermore, 84.1% of the samples had values less than or equal to 1 cfu/cm^2^ (value established as a limit in Decision 2001/471/EC, now repealed). These data are similar to those previously obtained by Alonso-Calleja et al. [27], in which only one of the samples from one of the two slaughterhouses studied (assuming 1.4% of the samples from that slaughterhouse) presented a value greater than 1 cfu/cm^2^.

The counts of coliforms (3.96 × 10^0^ ± 2.55 × 10^1^ cfu/cm^2^) were similar to those previously obtained by other authors in samples from poultry slaughterhouse surfaces (between 3.9 × 10^−1^ and 4.2 × 10^1^ cfu/cm^2^) [26]. The multiplication of these microorganisms may be affected by the storage at 4 °C of the samples, implying a lower value of their counts. However, some studies of raw milk [31] and poultry meat [32] suggested that this storage should be prolonged to a time above 24 h to be truly representative. In the study being presented here, microbiological analysis took place within a maximum period of 18 h from sample collection. In the case of enterococci, the average values in the present study ranged from 1.8 × 10^−1^ ± 7.79 × 10^−1^ cfu/cm^2^ (enterococci) to 1.12 × 10^−1^ ± 3.35 × 10^−1^ cfu/cm^2^ (VRE), which are much lower figures than those obtained by Nortjé et al. [29] from bovine slaughterhouse surfaces (between 7.9 × 10^3^ and 5.0 × 10^4^ cfu/cm^2^).

### 3.2. Isolation and Identification of Microorganisms

Among the isolations made from VRBGA (characteristic colonies of *Enterobacteriaceae* grew in 38.6% of the samples analyzed), *E. coli* was identified by MALDI-TOF and PCR in 47.1% of the positive samples (assuming 18.2% of the total samples analyzed), while the presence of *Salmonella* spp. was detected in none of them. The prevalence data for *E. coli* are lower than those obtained by other authors in pig (35.1%) [33] and bovine (41.6%) [34] slaughterhouses but higher than those found in lamb slaughterhouse surfaces (4.2%) [35]. Other authors obtained data similar to those of the present study, both for *E. coli* (20.2%) [36] and *Salmonella* spp. (0.34%) [37], but higher as for *Enterobacteriaceae* in general (60%) [38], on different surfaces of slaughterhouses and retail establishments. Monitoring the presence of different genera of *Enterobacteriaceae*, including the ones reported in this study, along the slaughter line enables the verification of slaughter operation hygiene and the use of good manufacturing practices by slaughter operators [39].

The prevalences of *Enterococcus* spp. obtained in this study (36.4%) are very different from those found by other authors consulted. Soares-Santos et al. [40] identified *E. faecium* strains in 75.0% of the swine slaughterhouse surface samples analyzed; Lavilla Lerma et al. [35] also detected strains of *Enterococcus* spp. on lamb slaughterhouse surfaces, but in a much smaller percentage (0.8%), and Wambui et al. [41] isolated strains of several *Enterococcus* species (*E. faecalis*, *E. mundtii*, *E. thailandicus*, *E. faecium*, *E. hirae*, *E. casseliflavus*, and *E. devriesei*) from slaughterhouse surfaces of small- and medium-sized enterprises in Kenya (assuming 26.6% of the samples analyzed).

It should be noted that vancomycin-resistant enterococci (VRE), confirmed by PCR at the genus level, were detected in five samples. VRE are associated with increasing frequency in both nosocomial and community-acquired human infections. Although there is little evidence that these infections are directly related to the consumption or handling of contaminated food, it is a proven fact that the presence of these microorganisms in food presents a clear risk of transferring resistance genes to other bacterial cells throughout the food chain, thereby contributing to the spread and persistence of antibiotic resistance in the food production chain [42,43].

Meanwhile, *Listeria* spp. was only detected in two samples (representing 4.6% of the total). The prevalence of *Listeria* is similar to the values obtained by other authors in samples collected from beef slaughterhouse surfaces, as follows: 6.0% [44], 5.6% (identified as *L. monocytogenes*, *L. innocua* and *L. ivanovii*) [45], and 4.9% of the samples in poultry slaughterhouses [37]. The contamination with these pathogenic bacteria when slaughtering animals may occur by means of direct fecal material, contaminated skin during the removal of viscera, and the contact of carcasses with one another, but also with the equipment in the slaughterhouse. For this reason, food of animal origin has become an important vehicle for the transmission of *Listeria* to humans [46].

### 3.3. Phenotypic Resistance to Antibiotics

The antibiotic resistance data obtained for *Enterobacteriaceae* were different, depending on whether it was *E. coli* or another *Enterobacteriaceae*. The *E. coli* isolates tested in the present study showed ranges of resistance (if taking in count both resistance and reduced susceptibility) to antibiotics similar to those previously obtained by Gregova et al. [47] in strains of this microbial species isolated in poultry slaughterhouses, as follows: 89.0% of the strains were resistant to ampicillin and 10.0% were resistant to chloramphenicol. Our results for ampicillin are also like those obtained by Elabbasy et al. [48] in strains isolated in beef slaughterhouse, (where 60% were resistant) but lower for other antibiotics: 65.0% were resistant to nalidixic acid, 55.0% were resistant to sulfamethoxazole/trimethoprim, and 75.0% were resistant to tetracycline.

For the rest of the enterobacteria, the results obtained in the present study were very different from those of *E. coli*, with lower resistance percentages. Contrarily, some authors have found very high resistance values for other species of *Enterobacteriaceae* from inert slaughterhouse surfaces. In this regard, Savin et al. [49] resistance in 96.7% (ciprofloxacin and cefotaxime) of *Klebsiella* spp. strains isolated from poultry slaughterhouse samples. Homeier-Bachmann et al. [50] obtained enterobacteria strains with high percentages of resistance to second and third generation cephalosporins (89% to cefotaxime and 95.0% to ceftazidime), and ciprofloxacin (53.0%).

In general, the enterococcal isolates analyzed showed a degree of resistance to antibiotics similar to that observed in other studies. Soares-Santos et al. [40] showed that all enterococcal isolates had sensitivity to ampicillin, ciprofloxacin, gentamicin, linezolid, nitrofurantoin, and teicoplanin, while 58% of the strains were resistant to quinupristin-dafopristin, 55% were resistant to tetracycline, 38% were resistant to rifampicin, 31% were resistant to erythromycin, 7% were resistant to streptomycin, 4% were resistant to chloramphenicol, and 1% were resistant to vancomycin. Similar results were obtained by Wambui et al. [41], who observed that all enterococcal isolates were sensitive to ciprofloxacin, penicillin, ampicillin, vancomycin, nitrofurantoin, teicoplanin, linezolid, and levofloxacin while presenting resistance to rifampicin (46.3%), erythromycin (23.9%), tetracycline (20.9%), and chloramphenicol (7.5%). Furthermore, it should be noted that the colonies isolated from the SBV medium, all of which were resistant to vancomycin (VRE), presented a higher percentage of resistance to antibiotics than those from the KAE medium.

Some of the antibiotics associated with the highest percentage of resistance or with reduced susceptibility strains, such as ampicillin, ciprofloxacin, streptomycin, cefoxitin, gentamycin, nalidixic acid, or tetracycline for *Enterobacteriaceae*, and streptomycin, gentamycin, kanamycin, tetracycline, or vancomycin for *Enterococcus*, are classified as “critically important” or “highly important” antimicrobial agents in human and veterinary medicine [51,52].

### 3.4. Detection of Resistance Genes to Beta-Lactam Antibiotics in Enterobacteriaceae

In this study, the presence of five resistance genes related to beta-lactam antibiotics (*bla_TEM_*, *bla_CTX-M_*, *bla_KPC_*, *bla_CYM-2_*, and *bla_NDM_*) was studied. The resistance mechanisms associated with these genes are based on the synthesis of extended-spectrum beta-lactamases capable of hydrolyzing all penicillins, cephalosporins, and carbapenems (with the exception of aztreonam). Furthermore, microorganisms carrying these genes usually have associated resistance mechanisms to other antibiotics, such as aminoglycosides or fluoroquinolones, leaving few therapeutic options available [53]. The use of carbapenems in veterinary medicine is off-label and should be reserved for cases with limited therapeutic alternatives and in which animals have a high likelihood of survival with appropriate therapy [54]. In Spain and the European Union, these drugs are not commonly used in agriculture or at the farm level to treat animals, but the localization of these genes in mobile genetic elements, together with others (conferring resistance to other beta-lactams, fluoroquinolones, or aminoglycosides), contributes significantly to its spread [55].

The prevalence of some of these genes was very high in *E. coli* agreeing with the fact that poultry meat is the meat that usually presents the greatest contamination with bacteria carrying extended-spectrum beta-lactamases [56]. The genes with the greatest presence in all the *Enterobacteriaceae* strains studied were *bla_TEM_* and *bla_NDM_*. However, their percentages of appearance were much lower than those found in poultry farms in China, with the detection ranges of these genes somewhere between 53.2% and 98.6% of the samples [57]. Although the prevalence of ESBLs tends to differ among countries, *bla_TEM_*, *bla_SHV_*, *bla_CTX-M_*, and *bla_OXA_* genes are the main classes of ESBLs and *bla_KPC_*, *bla_IMP_*, *bla_VIM_*, and *bla_NDM_* genes are the most common carbapenemases found in Gram-negative bacteria [55].

The presence of some of these genes has been previously reported by other authors in food-producing environments, particularly in poultry slaughterhouses. Lim et al. [58] characterized strains of *E. coli*-producing ESBL isolated from chicken slaughterhouses in South Korea. Wei et al. [59] studied the presence of plasmid-mediated ESBL in strains of *E. coli* that were also isolated from a chicken slaughterhouse in Korea. Adel et al. [60] found a high prevalence of ESBL resistance genes in *Salmonella* strains isolated from retail meats and slaughterhouses in Egypt. There have also been many studies about the presence of ESBL-producing strains isolated from slaughterhouses carried out in Spain and Europe, but most of them are focused on *Enterobacteriaceae* strains isolated from fresh meat or carcasses [61,62,63,64] instead of from strains isolated directly from the processing surfaces.

The frequency of CTX-M type beta-lactamases was considerably low (only 5.1% in the total of the isolates studied) compared to the data obtained by other authors, who have detected this type of gene in much higher percentages, as follows: 72.5% [65] and 100% [66]. This fact is also striking because some authors have shown that poultry meat is the origin of the plasmids that code for beta-lactamases of this type [67]. The CTX-M is a plasmid-encoded plasmid carrying resistance genes for other antibiotics, such as tetracycline, aminoglycosides, and sulfamethoxazole/trimethoprim [68]. This is a fact that can be observed in some of the isolates found in this study. One of the CTX-M-positive strains are sensitive to cefotaxime but resistant or with reduced susceptibility to other antibiotics from those groups, such as kanamycin, streptomycin, or tetracycline (Table S1).

Something similar occurs with the presence of TEM-type beta-lactamases, which have been detected in 27.1% of the isolates in the present study. Other authors have described this type of beta-lactamase as one of the most predominant in strains of *Salmonella* spp. isolated from poultry, poultry meat, and clinical samples from patients [69]. Furthermore, Gundran et al. [65] detected *bla_TEM_* genes in 58.0% of samples obtained from Philippine poultry farms.

In this study, carbapenemases genes were found throughout almost the entire slaughter chain. The *bla_TEM_* and *bla_NDM_* genes were the most common, being found in approximately at 50.0% strains of *Escherichia* isolated from different surfaces, such as the slaughter area, the stunning tank, different cutters, or aspiration equipment (Table S1). On the other hand, the *bla_KPC_* was the least predominant of the carbapenemases genes, being found in just some of the strains isolated in the cutting areas of the chain. This can occur as a result of the intense protocols of cleaning and disinfection applied throughout the first steps of the slaughter process and as a result of disinfectants effective against co- and cross-resistance, since corresponding genes are often located on the same plasmid [70].

## 4. Material and Methods

### 4.1. Sampling

Samples were taken in two poultry slaughterhouses (A and B) located in the northwest of Spain. Sampling was carried out on 22 different surfaces along the slaughter chain of each slaughterhouse (44 in total), from the animal stunning area to the cutting room (Table 3).

Sampling was carried out within a maximum of one hour after cleaning and disinfection and consisted of vigorously rubbing the surface with three swabs moistened with phosphate-buffered saline (PBS). The swabs were subsequently placed in tubes with 10 mL of PBS and stored at 4 °C until they were processed in the laboratory (which took place within a maximum period of 18 h from sample collection).

### 4.2. Microbiological Analysis

The levels of viable aerobic microbiota (total viable counts, TAC), psychrotrophic microorganisms, enterobacteria, coliforms, enterococci, and vancomycin-resistant enterococci (VRE) were determined through direct plate counts using specific media for each microbial group. Data were expressed as cfu/cm^2^. Because large areas were sampled, and bacteria levels were sometimes low, the results (cfu/cm^2^) in some cases are expressed as 10 to the power of a negative number. The prevalence of *Listeria* spp. (UNE-EN ISO 11290-1:2018) [71] and *Salmonella* spp. (UNE-EN ISO 6579-1:2017) [72] were determined, carrying out two stages of the enrichment of the samples in a liquid medium and subsequent seeding in a chromogenic medium. All culture media used (Oxoid Ltd., Basingstoke, United Kingdom), along with their incubation conditions, are listed in Table 4.

Colonies were isolated from all the samples presumed positive for any of the microbial groups studied (except TAC and psychrotrophs) with morphology and characteristics typical of the microorganism in question (enterobacteria, coliforms, enterococci, VRE, *Salmonella* spp. or *Listeria* spp.) for subsequent identification using MALDI-TOF and PCR. The isolated colonies were cultured at 37–42 °C (depending on the microbial group) for 24 h in test tubes with 9 mL of tryptone soya broth (TSB, Oxoid) and subsequently stored at −30 °C in TSB with 20% of glycerol.

### 4.3. Identification of Isolates Using MALDI-TOF

All isolates were streaked on tryptone soya agar (TSA, Oxoid) plates and cultured at 37 °C for 24 h for subsequent identification using matrix-assisted laser desorption ionization time-of-flight (MALDI-TOF). An isolated colony was taken from each sample using a sterile toothpick and distributed in a thin film on a well of a stainless-steel plate (Bruker Daltonics GmbH & Co. KG, Bremen, Germany). It was then air dried for 5 min and covered with 1 μL of 70% formic acid (Panreac, Barcelona, Spain). Once the acid had evaporated (approximately 5 min), the film was covered with 1 μL of HCCA matrix (α-Cyano-4-hydroxycinnamic acid; Bruker Daltonics) and allowed to dry for another 5 min at room temperature. Spectra were acquired with the MALDI Biotyper system and compared to the reference database (Bruker Daltonics).

### 4.4. Confirmation of Isolates Using PCR

The identification of the isolates (up to family, genus, or species level) was confirmed using polymerase chain reaction (PCR). To do so, genomic DNA was extracted from isolates grown in TSB (Oxoid) following the previously described protocol [77].

For the amplification reaction, 5 µL of the sample and 20 µL of the master mix, prepared with 1X NH4 Buffer, 2 mM MgCl_2_, 0.2 mM dNTPs (each), 0.5 mM primers (each one), 1U of Taq-DNA polymerase, and 14.25 µL of MiliQ water, were used. All reagents were supplied by BIORON GmbH (Ludwigshafen, Germany), except for dNTPs, which were purchased from EURX Sp. z o.o (Gdansk, Poland), and primers purchased from Macrogen (Beotkkot, Geumcheon-gu, Seoul, South Korea). The reactions were carried out in a ProFlex PCR System thermocycler (Waltham, MA, United States) programmed at 94 °C for 5 min, followed by the amplification cycles necessary for each pair of primers (Table 5) and 10 min of final elongation at 72 °C.

### 4.5. Phenotypic Resistance to Antibiotics

The susceptibility of the strains to some of the families of antibiotics most used in clinical practice was determined. These included aminoglycosides, beta-lactams, macrolides, glycopeptides, sulfonamides, rifamycins, tetracyclines, phenicols, fluoroquinolones, and nitrofurans. For this, the isolates were seeded in tubes with 9 mL of Mueller Hinton Broth (MHB, Oxoid) and incubated at 37 °C for 8–10 h, until they were in the exponential growth phase. Subsequently, antibiograms of each strain were performed (disk diffusion technique), sowing the grass cultures on Mueller Hinton Agar (MHA, Oxoid) plates. The plates were incubated at 37 °C for 18–20 h, after which time the inhibition zones were measured, allowing the strains to be classified as susceptible, with reduced susceptibility (intermediate), or resistant according to the established criteria [82].

The following antibiotic disks (Oxoid) were used for *Enterobacteriaceae* isolates: gentamicin (CN, 10 µg), kanamycin (K, 30 µg), streptomycin (S, 10 µg), amoxoxycillin/clavulanic acid (AMC, 30 µg), ampicillin (AMP, 10 µg), ceftazidime (CAZ, 30 µg), cefotaxime (CTX, 30 µg), cefoxitin (FOX, 30 µg), meropenem (MEM, 10 µg), aztreonam (ATM, 30 µg), chloramphenicol (C, 30 µg), ciprofloxacin (CIP, 5 µg), nalidixic acid (NA, 30 µg), sulfamethaxazole/trimethoprim (SXT, 25 µg), fosfomycin (FOS, 50 µg) and nitrofurantoin (F, 300 µg). While for *Enterococcus* spp. isolates the used disks were: ampicillin (AMP, 10 µg), chloramphenicol (C, 30 µg), erythromycin (E, 15 µg), fosfomycin (FOS, 50 µg), quinupristin/dalfopristin (15 µg), linezolid (LZD, 10 µg), nitrofurantoin (F, 300 µg), rifampicin (RD, 5 µg), teicoplanin (TEC, 30 µg), tetracycline (TE, 30 µg), and vancomycin (VA, 30 µg).

### 4.6. Detection of Resistance Genes to Beta-Lactam Antibiotics in Enterobacteriaceae

The production capacity of five of the most common extended-spectrum beta-lactamases was determined using real-time PCR (qPCR) detection of the *bla_TEM_*, *bla_CTX-M-15_*, *bla_KPC_*, *bla_CYM-2_*, and *bla_NDM_* genes (Table 6). For the amplification reaction, 6.5 µL of the previously extracted genomic DNA (10 ng/µL), 10 µL of the commercial Forget-Me-Not™ EvaGreen^®^ qPCR master mix (2×; Biotium, Landing Parkway, Fremont, CA, USA), 3 µL of Rox (1:10; Biotium), and 0.25 µL of each primer (25 µM; Macrogen) were used. The reactions were carried out in a StepOne™ thermocycler (Applied Biosystems, Foster City, CA, USA) programmed with an initial denaturation at 95 °C for 10 min, followed by 40 cycles made up of 5 s at 95 °C and 30 s at 60 °C. At the end of the amplification, a melting curve was performed using the following cycling parameters: 57 °C for 30 s and temperature changes of 5 °C to a final temperature of 99 °C. Each PCR reaction included a negative control (without DNA).

### 4.7. Statistical Analysis

After verifying that the data did not meet the normality (Shapiro–Wilk test) and homogeneity of variances (Levene test) criteria, microbial counts were analyzed using the Kruskal–Wallis test. To compare microbial groups (pairwise) and slaughterhouses (A and B), Mann–Whitney tests were performed. All analyses were carried out using RStudio 4.1.3 software [84], establishing a probability level of 95% to determine significant differences (*p* < 0.05).

## 5. Conclusions

The results obtained in this research allow us to describe the microbiota present on the surfaces and equipment of different areas of the slaughter line (from stunning to cutting) of two poultry slaughterhouses. The average levels of microorganisms indicating hygienic quality (TAC, psychrotrophs, enterobacteria, coliforms, and enterococci) are in the range of values obtained by other authors, although important differences have been found between surfaces. The high presence of strains with resistance to some antibiotics and, in some cases, the high prevalence of genes for resistance to beta-lactams is striking. This fact highlights the importance of establishing appropriate measures to control the spread of microorganisms resistant to antibiotics in poultry slaughterhouses. However, the low prevalence of potentially pathogenic species, such as *Salmonella* spp. and *Listeria* spp., and their low level of resistance to the antibiotics most used for the treatment of these infections is reassuring. These findings are of great practical importance since they can serve as a basis for improving cleaning and disinfection procedures in poultry slaughterhouses and other similar meat industries. It should be noted, however, that data from only two slaughterhouses are presented, so additional studies should be conducted to obtain more general conclusions.

## Figures and Tables

**Table 1:** Load (cfu/cm^2^) of the different microbial groups analyzed.

Sample	TAC	Psychrotrophs	Enterobacteria	Coliforms	Enterococci	VRE
A1	4.25 × 10^1^	3.33 × 10^1^	1.22 × 10^0^	5.47 × 10^−1^	1.50 × 10^−1^	1.13 × 10^−1^
A2	4.18 × 10^2^	3.36 × 10^2^	1.14 × 10^−1^	9.37 × 10^−1^	3.12 × 10^−1^	<5.20 × 10^−2^
A3	1.41 × 10^2^	8.49 × 10^1^	<3.92 × 10^−3^	<3.92 × 10^−3^	7.84 × 10^−3^	<1.96 × 10^−2^
A4	9.55 × 10^2^	2.51 × 10^2^	<1.25 × 10^−2^	<1.25 × 10^−2^	<1.25 × 10^−2^	<6.25 × 10^−2^
A5	1.74 × 10^0^	1.61 × 10^−1^	<1.46 × 10^−3^	<1.46 × 10^−3^	<1.46 × 10^−3^	<7.31 × 10^−3^
A6	2.38 × 10^3^	1.34 × 10^3^	<5.70 × 10^−2^	1.14 × 10^−1^	1.88 × 10^0^	<2.85 × 10^−1^
A7	2.12 × 10^2^	1.90 × 10^2^	7.47 × 10^−1^	1.68 × 10^−2^	7.47 × 10^−2^	4.67 × 10^−2^
A8	3.12 × 10^1^	1.63 × 10^1^	<2.21 × 10^−3^	<2.21 × 10^−3^	<2.21 × 10^−3^	<1.11 × 10^−2^
A9	9.07 × 10^1^	5.76 × 10^1^	<5.71 × 10^−3^	<5.71 × 10^−3^	<5.71 × 10^−3^	<2.86 × 10^−2^
A10	4.80 × 10^1^	3.66 × 10^1^	1.57 × 10^−3^	<1.57 × 10^−3^	<1.57 × 10^−3^	<7.86 × 10^−3^
A11	4.94 × 10^0^	1.70 × 10^0^	<7.72 × 10^−3^	<7.72 × 10^−3^	7.72 × 10^−3^	2.16 × 10^0^
A12	1.16 × 10^2^	9.31 × 10^1^	1.29 × 10^1^	1.73 × 10^0^	2.67 × 10^−2^	<3.33 × 10^−2^
A13	1.58 × 10^1^	9.19 × 10^0^	3.23 × 10^−2^	<4.04 × 10^−3^	<4.04 × 10^−3^	<2.02 × 10^−2^
A14	4.29 × 10^2^	2.91 × 10^2^	2.10 × 10^−1^	<3.62 × 10^−3^	<3.62 × 10^−3^	<1.81 × 10^−2^
A15	1.60 × 10^1^	1.28 × 10^1^	<3.43 × 10^−3^	<3.43 × 10^−3^	<3.43 × 10^−3^	<1.72 × 10^−2^
A16	1.00 × 10^1^	6.00 × 10^0^	<2.58 × 10^−3^	<2.58 × 10^−3^	<2.58 × 10^−3^	<1.29 × 10^−2^
A17	9.79 × 10^1^	1.93 × 10^2^	5.84 × 10^0^	<4.87 × 10^−3^	<4.87 × 10^−3^	<2.43 × 10^−2^
A18	1.72 × 10^1^	2.96 × 10^0^	<3.69 × 10^−3^	<3.69 × 10^−3^	<3.69 × 10^−3^	<1.85 × 10^−2^
A19	4.17 × 10^2^	4.59 × 10^2^	<3.06 × 10^−2^	<3.06 × 10^−2^	<3.06 × 10^−2^	<1.53 × 10^−1^
A20	3.87 × 10^1^	3.24 × 10^1^	2.01 × 10^−1^	3.92 × 10^−2^	<4.90 × 10^−3^	<2.45 × 10^−2^
A21	3.45 × 10^2^	1.05 × 10^2^	3.30 × 10^0^	<2.82 × 10^−3^	2.82 × 10^−3^	<1.41 × 10^−2^
A22	5.48 × 10^2^	1.16 × 10^3^	1.95 × 10^1^	<5.17 × 10^−3^	1.55 × 10^−2^	<2.58 × 10^−2^
B1	7.49 × 10^1^	1.33 × 10^1^	<1.02 × 10^−2^	<1.02 × 10^−2^	<1.02 × 10^−2^	<5.12 × 10^−2^
B2	2.44 × 10^2^	8.95 × 10^1^	1.06 × 10^−2^	3.17 × 10^−2^	<4.23 × 10^−2^	<5.29 × 10^−2^
B3	5.23 × 10^1^	1.94 × 10^1^	<6.76 × 10^−3^	<6.76 × 10^−3^	<6.76 × 10^−3^	<3.38 × 10^−2^
B4	4.70 × 10^2^	1.95 × 10^2^	<1.29 × 10^−2^	<1.29 × 10^−2^	<3.87 × 10^−2^	<6.45 × 10^−2^
B5	6.14 × 10^0^	5.39 × 10^−1^	1.46 × 10^−3^	<1.46 × 10^−3^	<1.46 × 10^−3^	<7.28 × 10^−3^
B6	5.05 × 10^2^	2.48 × 10^1^	7.07 × 10^−1^	1.06 × 10^0^	<2.65 × 10^−1^	<4.42 × 10^−1^
B7	6.21 × 10^0^	6.28 × 10^−1^	<4.57 × 10^−3^	<4.57 × 10^−3^	<4.57 × 10^−3^	<2.28 × 10^−2^
B8	4.11 × 10^0^	4.28 × 10^0^	<4.57 × 10^−3^	<4.57 × 10^−3^	<4.57 × 10^−3^	<2.28 × 10^−2^
B9	8.70 × 10^1^	1.71 × 10^−1^	<5.71 × 10^−3^	<5.71 × 10^−3^	<5.71 × 10^−3^	<2.85 × 10^−2^
B10	1.18 × 10^2^	6.18 × 10^1^	4.01 × 10^−1^	2.13 × 10^−2^	<3.04 × 10^−3^	<1.52 × 10^−2^
B11	1.27 × 10^3^	2.90 × 10^3^	8.91 × 10^1^	1.69 × 10^2^	4.88 × 10^0^	5.71 × 10^−1^
B12	1.43 × 10^1^	8.84 × 10^0^	<3.24 × 10^−2^	<3.24 × 10^−2^	<3.24 × 10^−2^	<1.62 × 10^−1^
B13	8.97 × 10^2^	5.72 × 10^2^	<1.20 × 10^−2^	<1.20 × 10^−2^	<1.20 × 10^−2^	<5.98 × 10^−2^
B14	1.80 × 10^2^	9.91 × 10^1^	1.63 × 10^0^	4.18 × 10^−1^	<9.09 × 10^−3^	<4.55 × 10^−2^
B15	1.91 × 10^1^	1.75 × 10^1^	4.56 × 10^−2^	9.12 × 10^−3^	<3.04 × 10^−3^	<1.52 × 10^−2^
B16	8.76 × 10^1^	3.45 × 10^−2^	<6.90 × 10^−3^	<6.90 × 10^−3^	<6.90 × 10^−3^	<3.45 × 10^−2^
B17	7.59 × 10^1^	1.93 × 10^−1^	<5.14 × 10^−3^	<5.14 × 10^−3^	<5.14 × 10^−3^	<2.57 × 10^−2^
B18	3.60 × 10^1^	1.81 × 10^1^	2.27 × 10^−2^	5.67 × 10^−3^	<5.67 × 10^−3^	<2.83 × 10^−2^
B19	1.46 × 10^1^	7.31 × 10^−2^	<2.66 × 10^−3^	<2.66 × 10^−3^	<2.66 × 10^−3^	<1.33 × 10^−2^
B20	2.43 × 10^0^	1.91 × 10^−1^	6.93 × 10^−3^	2.77 × 10^−2^	6.93 × 10^−3^	3.47 × 10^−2^
B21	1.16 × 10^0^	2.00 × 10^−1^	<7.26 × 10^−3^	<7.26 × 10^−3^	<7.26 × 10^−3^	<3.63 × 10^−2^
B22	3.63 × 10^0^	2.79 × 10^1^	<5.01 × 10^−3^	<5.01 × 10^−3^	<5.01 × 10^−3^	<2.51 × 10^−2^

TAC, total aerobic counts; VRE, vancomycin-resistant enterococci. Slaughterhouse A: A1, stunning tank; A2, slaughter area; A3, bleeding tank; A4, sink (outer surface); A5, scalding tank door; A6, plucker (rubber fingers); A7, cloacal sphincter splitter; A8, abdominal opening device; A9, eviscerator; A10, abdominal cavity aspiration device; A11, trachea removal device; A12, neck cutter; A13, spray washer; A14, leg and neck conveyor belt; A15, sorter; A16, box conveyor belt; A17, cutting board; A18, chopper; A19, kneader; A20, stuffer; A21, meat conveyor belt; and A22, packaging area cutting board. Slaughterhouse B: B1, stunning tank; B2, slaughter area; B3, bleeding tank; B4, sink (outer surface); B5, scalding tank door; B6, plucker (rubber fingers); B7, cloacal sphincter splitter; B8, abdominal opening device; B9, eviscerator; B10, leg cutter; B11, head cutter; B12, gizzard conveyor belt; B13, crop extractor; B14, neck-bone cutter; B15, abdominal aspiration device; B16, leg conveyor belt; B17, liver conveyor belt; B18, pre-cooling rollers; B19, container for collecting chicken wings; B20; breast conveyor belt; B21, thigh conveyor belt; and B22, packaging area cutting board.

**Table 2:** Surfaces and culture media from which the colonies analyzed were isolated in the two slaughterhouses (A and B).

Sample	Gram-Negative Bacteria		Gram-Positive Bacteria	
	*Enterobacteriaceae*	*E. coli*	*Enterococcus* spp.	*Listeria* spp.
A1		VRBGA/VRBA/SCA	KAE/SBV	
A2		VRBGA/VRBA/SCA	KAE	
A3			KAE	
A4		VRBGA		
A6	VRBGA/VRBA		KAE	
A7		VRBGA/VRBA	KAE/SBV	
A10		VRBGA		
A11			SBV	
A12	VRBGA/VRBA		KAE	
A13	VRBGA			OCLA
A14	VRBGA			
A17	VRBGA			
A20	VRBGA/VRBA			
A21	VRBGA		KAE	
A22	VRBGA/SCA		KAE	OCLA
B2		VRBGA/VRBA	KAE	
B3			KAE	
B4	SCA		KAE	
B5	VRBGA			
B6		VRBGA/VRBA	KAE	
B10	VRBGA	VRBA		
B11		VRBGA/VRBA	SBV	
B14	VRBGA/VRBA			
B15	VRBGA	VRBA/SCA		
B18	VRBGA/VRBA			
B20	VRBA		SBV	

VRBGA, violet red bile glucose agar; VRBA, violet red bile agar; KAE, kanamycin aesculin azide agar; SBV, Slanetz and Bartley agar, supplemented with vancomycin; OCLA, chromogenic *Listeria* agar; SCA, chromogenic *Salmonella* agar. A total of 63 isolates were taken as follows: 1 isolate from each sample and culture medium in the case of Enterobacteriaceae different from *E. coli* (21 isolates), *E. coli* (19), and *Enterococcus* spp. (17), and 3 isolates from each sample (2) in the case of *Listeria* spp. For additional interpretation, see Table 1.

**Table 3:** Surfaces analyzed and area (cm^2^) of each of them.

Slaughterhouse A			Slaughterhouse B		
Identification	Surface	Area (cm^2^)	Identification	Surface	Area (cm^2^)
A1	Stunning tank	3544.5	B1	Stunning tank	976.0
A2	Slaughter area	960.8	B2	Slaughter area	945.0
A3	Bleeding tank	2550.0	B3	Bleeding tank	1480.0
A4	Sink (outer surface)	800.0	B4	Sink (external surface)	775.0
A5	Scalding tank door	6842.5	B5	Scalding tank door	6870.0
A6	Plucker (rubber fingers)	175.3	B6	Plucker (rubber fingers)	113.1
A7	Cloacal sphincter splitter	5351.5	B7	Cloacal sphincter splitter	2190.0
A8	Abdominal opening equipment	4515.8	B8	Abdominal opening equipment	2190.0
A9	Eviscerator	1750.8	B9	Eviscerator	1752.0
A10	Abdominal cavity aspirator equipment	6360.3	B10	Leg cutter	3294.0
A11	Trachea removal equipment	1295.8	B11	Head cutter	1050.0
A12	Neck cutter	1500.0	B12	Gizzard conveyor belt	308.2
A13	Spray washing equipment	2475.0	B13	Crop extractor	836.0
A14	Leg and neck carrying belt	2760.0	B14	Bone neck cutter	1100.0
A15	Sorter	2915.0	B15	Abdominal aspiration equipment	3290.0
A16	Box conveyor belt	3876.7	B16	Leg conveyor belt	1449.0
A17	Cutting board	2054.4	B17	Liver conveyor belt	1944.0
A18	Chopper	2707.3	B18	Pre-cooling rollers	1764.0
A19	Kneader	326.7	B19	Container for collecting chicken wings	3760.0
A20	Stuffer	2040.0	B20	Breast conveyor belt	1443.0
A21	Meat conveyor belt	3550.0	B21	Thigh conveyor belt	1378.0
A22	Packaging area cutting board	1935.0	B22	Packaging area cutting board	1995.0

**Table 4:** Culture media and incubation conditions used for the determination of each microbial group.

Microorganism	Culture Media	Incubation (T/t)	Reference
TAC ^1^	PCA	30 °C/72 h	[73]
Psychrotrophs ^1^	PCA	7 °C/10 days	[74]
Enterobacteria ^2,3^	VRBGA	37 °C/24 h	[75]
Coliforms ^2,3^	VRBA	42 °C/24 h	[75]
Enterococci ^2^	KAE	42 °C/24 h	[75]
Vancomycin-resistant enterococci ^1^	SBV	42 °C/48 h	[76]
*Listeria* spp.	Semi-Fraser broth	30 °C/24 h	
	Fraser broth	37 °C/24 h	UNE-EN ISO 11290-1:2018 [71]
	OCLA	37 °C/48 h	
*Salmonella* spp.	Buffered peptone water	37 °C/24 h	
	Rappaport-Vassiliadis broth	42 °C/24 h	UNE-EN ISO 6579-1:2017 [72]
	SCA	42 °C/24 h	

TAC, total aerobic counts; PCA, plate count agar; VRBGA, violet red bile glucose agar; VRBA, violet red bile agar; KAE, kanamycin aesculin azide agar; SBV, Slanetz and Bartley agar, supplemented with vancomycin; OCLA, chromogenic *Listeria* agar; SCA, chromogenic *Salmonella* agar. ^1^, spread plate method (0.1 mL); ^2^, pour plate method (1 mL); ^3^, overlay procedure. All media were obtained from Oxoid (Hampshire, UK), with the exception of SCA (Sigma-Aldrich, St. Louis, MI, USA).

**Table 5:** Primers used for each microorganism with its nucleotide sequence, the size of the amplified fragment (bp), and the thermocycling program used.

Microorganism	Primer	Sequence (5′ to 3′)	Size	Program
*Enterobacteriaceae*	rpoB-F	CAGGTCGTCACGGTAACAAG	512 pb	(94 °C—30 s/60 °C—30 s/72 °C—60 s) × 35
	rpoB-R	GTGGTTCAGTTTCAGCATGTAC		
*Escherichia coli*	uidA-F	GTCACGCCGTATGTTATTG	530 pb	(94 °C—30 s/58 °C—60 s/72 °C—90 s) × 35
	uidA-R	CCAAAGCCAGTAAAGTAGAAC		
*Enterococcus* spp.	16S-F	TCAACCGGGGAGGGT	738 pb	(94 °C—30 s/60 °C—60 s/72 °C—45 s) × 30
	16S-R	ATTACTAGCGATTCCGG		
*Listeria* spp.	prs-F	GCTGAAGAGATTGCGAAAGAAG	370 pb	(94 °C—30 s/58 °C—30 s/72 °C—45 s) × 35
	prs-R	CAAAGAAACCTTGGATTTGCGG		

The primer sequences were obtained from Fazzeli et al. [78] for the identification of *Enterobacteriaceae*, from El-Sayed et al. [79] for *Escherichia coli*, from Deasy et al. [80] for *Enterococcus* spp., and from Ryu et al. [81] for *Listeria* spp.

**Table 6:** Primers used for qPCR detection of the beta-lactam resistance genes studied.

Gene	Primer	Sequence(5′ to 3′)	Size	Reference
*bla_TEM_*	F	GATAAATCTGGAGCCGGTGA	78 pb	[16]
	R	GATACGGGAGGGCTTACCAT		
*bla_CTX-M_*	F	CACCAATGATATTGCGGTGA	77 pb	[16]
	R	GTTGCGGCTGGGTAAAATAG		
*bla_KPC_*	F	CTGTATCGCCGTCTAGTTCTG	101 pb	[83]
	R	AGTTTAGCGAATGGTTCCG		
*bla_CMY-2_*	F	CCAGAACTGACAGGCAAACA	65 pb	[16]
	R	CCTGCCGTATAGGTGGCTAA		
*bla_NDM_*	F	ATGGAGACTGGCGACCAAC	87 pb	[16]
	R	GGCATGTCGAGATAGGAAGG		

## Data Availability

The raw data supporting the conclusions of this article will be made available by the authors on request.

## Supplementary Materials

The following supporting information can be downloaded at https://www.mdpi.com/article/10.3390/antibiotics13070587/s1: Table S1: Results of antimicrobial susceptibility tests (disk diffusion) and detection of antibiotic resistance genes (PCR).
